# Detecting functional change in response to exercise in knee osteoarthritis: a comparison of two computerized adaptive tests

**DOI:** 10.1186/s12891-018-1942-9

**Published:** 2018-01-23

**Authors:** Feng-Hang Chang, Alan M. Jette, Mary D. Slavin, Kristin Baker, Pengsheng Ni, Julie J. Keysor

**Affiliations:** 1 Graduate Institute of Injury Prevention and Control, College of Public Health, Taipei Medical University, 250 Wu-Hsing Street, Taipei City, 11031 Taiwan; 2 MGH Institute of Health Professions, 36 1st Avenue, Charlestown Navy Yard, Boston, MA 02129-4557 USA; 3 Health Law, Policy and Management, Boston University School of Public Health, 715 Albany St., 5th floor West, Boston, MA 02118 USA; 4 Boston University College of Health and Rehabilitation Sciences: Sargent College, Boston, USA; 5 Health Law, Policy and Management, Boston University School of Public Health, 715 Albany St., 5th floor West, Boston, MA 02118 USA; 6 MGH Institute of Health Professions, Department of Physical Therapy, 36 1st Avenue, Boston, MA 02129 USA

**Keywords:** Osteoarthritis, Measurement, Patient reported outcomes, Computerized adaptive testing

## Abstract

**Background:**

The intent of this study was to examine and compare the ability to detect change of two patient reported outcome (PRO) instruments that use a computerized adaptive test (CAT) approach to measurement. The Patient Reported Outcomes Measurement Information System (PROMIS®) Physical Function scale is a generic PRO, while the Osteoarthritis Computerized Adaptive Test (OA-CAT) is an osteoarthritis-specific PRO.

**Methods:**

This descriptive, longitudinal study was conducted in a community setting, involving individuals from the greater Boston area. Inclusion criteria: age > 50, self-reported doctor-diagnosed knee osteoarthritis (OA) and knee pain. The PROMIS® Physical Function CAT and OA-CAT Functional Difficulty scale were administered at baseline and at the conclusion of a 6-week exercise program. Effect sizes (ES) were calculated for both measures, and bootstrap methods were used to construct confidence intervals and to test for significant ES differences between the measures.

**Results:**

The OA-CAT Functional Difficulty scale achieved an ES of 0.62 (0.43, 0.87) compared to the PROMIS® Physical Function CAT ES of 0.42 (0.24, 0.63). ES estimates for the two CAT measures were not statistically different.

**Conclusions:**

The condition-specific OA-CAT and generic PROMIS® Physical Function CAT both demonstrated the ability to detect change in function. While the OA-CAT scale showed larger effect size, no statistically significant difference was found in the effect size estimates for the generic and condition-specific CATs. Both CATs have potential for use in arthritis research.

**Trial registration:**

This trial is registered with ClinicalTrials.gov on 6/21/11 (Identifier NCT01394874)

## Background

Function is a widely targeted area for osteoarthritis (OA) treatment, including pharmacological and non-pharmacological approaches. Patient reported outcomes (PROs) that assess quality of life such as decreased function are increasingly recognized as an important component of OA intervention outcome assessment. Numerous PRO measures are used in OA research [1–6], but a review of the psychometric properties of 32 OA outcome measures concluded that these measures are uniformly rated low in terms of their ability to detect change [1]. The ability of a measure to detect change is of utmost importance when considering which measure to use for research trials and to monitor clinical progress.

Traditional fixed-form PRO measures, such as the 36-Item Short Form Health Survey (SF-36) and the Western Ontario and McMaster Universities Osteoarthritis Index (WOMAC), are comprised of a fixed number of items that are administered to all participants. To reduce administration burden, measures are constrained to a limited number of items, often lacking coverage of the broad range of ability typically observed in patient populations [5–7]. This limitation of fixed-form PRO measures raises concerns about the loss of score precision and the reduced ability to measure clinically meaningful change [8, 9]. Furthermore, these measures require respondents to answer every question even though some may be redundant and inappropriate for an individual [10–12].

Item response theory (IRT) [13] and computerized adaptive testing (CAT) [14] are advances in measurement that have great potential to overcome limitations of fixed-form PRO measures and improve the ability to assess the wide range in functional ability seen in persons with OA. IRT-based measures use calibrated banks of items that are hierarchically organized from low to high ability for the outcome of interest. These banks of items can be administered as CATs, which employ computer-based algorithms to select items that match an individual’s ability based on his/her responses to previously administered items. Using this approach, a relatively small number of items (e.g., 4–10) can generate a precise estimate of the individual’s ability for a specific outcome domain. In this way, IRT/CAT measures can provide adequate measurement breadth, precision and sensitivity to change without being burdensome, characteristics that are important for measures used in research and clinical practice [15].

Two IRT/CAT measures are available for use in OA, one is disease-specific and one is generic. The Osteoarthritis Computerized Adaptive Test (OA-CAT) is an IRT/CAT measure developed specifically for use in OA [16, 17]. Disease-specific measures contain items, responses, or other aspects of the measurement that are developed specifically for the disease or condition being measured. In contrast, generic measures are designed to assess outcomes with the expectation that the measurement models can be applied across populations without regard to the patient’s specific condition [18]. The Patient Reported Outcomes Measurement Information System (PROMIS®), funded by the National Institutes of Health, was designed as a generic measure to promote comparability across studies and clinical diagnoses [19]. Several PROMIS® domains, including physical functioning [19], can be used to assess outcomes in persons with osteoarthritis.

Faced with measurement choices among emerging PROs for OA research, it is important to examine an instrument’s psychometric properties in the population of interest. Indeed, to date there is no sensitivity to change data among persons with OA for either the OA-CAT or PROMIS® instruments. The intent of this study, therefore, was to examine the ability of two PRO CATs – the generic PROMIS® Physical Function (PF) CAT v1.0 and the osteoarthritis-specific OA-CAT Functional Difficulty (FD) scale – to detect change in physical function in a sample of adults with knee OA engaged in an exercise program designed to improve physical function.

## Methods

### Design and participants

This study, part of a randomized controlled trial to examine the effects of an intervention to improve long-term exercise adherence, reports on data collected at baseline and at the conclusion of a 6-week exercise program designed to improve function in persons with OA. The sample included 120 persons with knee osteoarthritis. The two CAT PRO measures were administered to all participants prior to engaging in a 6-week structured exercise program (baseline) and again after the exercise program was completed (post-exercise training). The exercise program was the same for all participants. OA-CAT FD and PROMIS® PF CAT data reported in this study were collected prior to randomization into intervention and control groups for the exercise adherence study.

Participants were recruited from the greater Boston area. Inclusion criteria were age > 50 years with self-reported, doctor-diagnosed knee OA and knee pain, which was determined by answering yes to both “Have you had pain on most days of the previous month?” and “Have you had pain for most months of the past year?” Exclusion criteria included a medical condition precluding exercise (stroke or myocardial infarction in the past 3 months, treatment for cancer, severe systemic disease), a medical condition that limits physical function more than the knee pain (including back or hip pain more severe than knee pain), inflammatory arthritis, current regular exerciser, regular resistance training (one or more times per week) in the last 6 months, plan for knee replacement during the trial, dementia or inability to follow exercise instructions and use the exercise adherence intervention.

### Measures

Characteristics of study measures are summarized in Table 1. Study measures included two IRT-based PRO measures that use a CAT approach: the PROMIS® PF CAT v 1.0 scale and the OA-CAT FD scale. Development of PROMIS® measures was initiated by the National Institute of Health Roadmap for Medical Research to advance the science and application of PROs in chronic diseases [18, 20]. The PROMIS® PF CAT is linked to a U.S. general population and the scale has a mean of *T* = 50 with an SD of 10 [21]. The PROMIS® PF scale assesses one’s capability to perform a variety of physical activities, including mobility (lower extremity function), dexterity (upper extremity function), axial (neck and back function), and ability to carry out instrumental activities of daily living. The PROMIS® PF v 1.0 CAT was administered using the default stopping rule (minimum of 4 items and maximum of 12 items) or reaching a standard error of 0.40. The OA-CAT [16, 17] was developed to measure conceptually distinct dimensions of functional pain, functional difficulty, and disability that are relevant for OA clinical practice and research. OA-CAT was developed based on an osteoarthritis sample with a scale range from 10 (lowest score) to 90 (highest score). The OA-CAT stopping rule is a maximum number of items (10) or reaching a standard error < 0.25. Preliminary studies show that the OA-CAT has improved breadth, precision and reliability and reduced floor and ceiling effects compared with the WOMAC, a traditional fixed-form PRO instrument [16, 17]. In this study, the OA-CAT FD scale was compared to the PROMIS® PF CAT. The OA-CAT was developed and its psychometric properties were examined using an analytic approach similar to PROMIS, but there are a few differences. Instead of using Graded Response model (GRM), we calibrated the OA-CAT item bank using Generalized Partial Credit model (GPCM). Because those two models have exact the same number of parameters and usually data fits one of the model will also fit the other model [22], so different models should have little impact on calibrating the item bank. Second, we didn’t remove the local dependent items as PROMIS did, instead in the CAT program we treated those local dependent items as “enemy” items – the CAT program only allows to select one item within a set of locally dependent items. Third, we applied weighted maximum likelihood (WML) estimation method in estimating the person score not Expected A Posterioi (EAP) method. The studies have shown WML estimator had less bias than EAP estimator [23, 24].

**Table 1:** Summary of Measures

Measure (*N* = items)	Item Stem	Response Options
OA-CAT Item Bank Functional difficulty (*N* = 125)	Because of the arthritis in your legs, how much difficulty did you have on an average day, over the past month when….?	• None • A little • A lot • Did not do the activity because of the arthritis in his/her legs • Did not do the activity for reasons other than the arthritis in his/her legs
PROMIS® CAT Item Bank v1.0 Physical Function (*N* = 124)	Does your health now limit you in…?	• Not at all • Very little • Somewhat • Quite a lot • Cannot do
	Are you able to…?	• Without any difficulty • With a little difficulty • With some difficulty • With much difficulty • Unable to do

### Data analyses

*Descriptive analysis*: Descriptive analyses were performed on assessments completed at baseline and post-exercise training. Means and standard deviations (SD) were computed for each measure: PROMIS® PF CAT and OA-CAT FD.

*OA-CAT FD and PROMIS® PF comparability*: It is important to examine comparability when assessing ability of the measures to detect change. Four analyses were conducted to compare measures: 1.) Pearson correlations examined the degree to which the OA-CAT FD and PROMIS® PF CAT assess a similar construct; 2.) score reliability, calculated as [1-average (squared standard error/variance of score)]; 3.) score distributions; and 4.) the number of items administered and time to complete each instrument.

*Ability to detect change*: Change scores were calculated based on assessments completed at baseline and post-exercise training. The Cohen effect size (ES) was computed as a standardized indicator of the ability of each instrument to detect true change [25]. The ES expresses change scores in terms of the underlying sampling distribution using SD estimates [26], and was calculated as (M2-M1)/S_b_ [27]. M2 is the mean post-intervention score and M1 is the mean pre-intervention score of each assessment; S_b_ is the baseline SD. The larger values of ES indicate greater ability to detect change. An ES of 0.2 reflects small change, an ES of 0.5 reflects moderate change, and an ES of 0.8 reflects large change [27].

The bootstrap method was used to calculate the 95% confidence intervals (CI) for ES estimates of each measure and to test for significant differences in ES estimates of the two measures [27]. Five thousand bootstrap samples of the difference in ES estimates across the measures were generated and estimates of differences in ES values were rank-ordered, and the 95% CI for the differences in ES estimates between the measures was calculated.

## Results

Table 2 shows the baseline demographic characteristics of the 120 participants consented into the study; of these, 104 completed the exercise program for whom we had follow-up data. Demographic characteristics of the sample are not statistically different for those enrolled at baseline and those who completed the 6-week exercise program, except for the percent of the sample identified as obese, which was significantly lower for exercise program completers.

**Table 2:** Sample Demographics

	Mean (SD) or Frequency (%)	
Variable	Baseline Sample(*N* = 120)	Post-Exercise training Sample (*N* = [104), *p* value*
Age (years)	65.06 (SD = 7.39)	65.57(SD = 7.38), *P* = 0.05
Gender		
Female	95 (79.2%)	85(81.7%)
Male	25 (20.8%)	19(18.3%), *P* = 0.1
Race		
White, Non-Hispanic	70 (58.3%)	62(59.6%)
Black, Non-Hispanic	33 (27.5%)	28(26.9%)
Hispanic	4 (3.3%)	2(1.9%)
Others	13 (10.8%)	12(11.6%), *p* = 0.1494
Body Mass Index (BMI)		
Underweight (< 18.50)	1 (0.83%)	1(0.96%)
Normal range (18.50–24.99)	24 (20%)	22(21.15%)
Overweight (25.00–29.99)	36 (30.0%)	28(26.92%)
Obese (> = 30.00)	59 (49.17%)	53(50.96%), *P* = 0.1587**
Knee replacement		
Yes	12 (10.1%)	9(8.7%)
No	107 (89.2%)	94(90.4%)
Missing	1(0.8%)	1(1%), *P* = 0.2042

*: *p* value of testing the demographic variables for subjects with and without Post-Exercise training data

**: categories (underweight and normal range) were merged; categories (overweight and obese) were merged

Note: BMI is calculated by body weight divided by the square of the height

### Instrument comparability

**Fig. 1:**
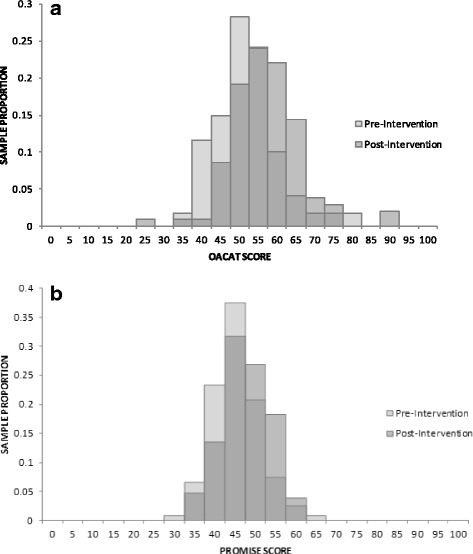
**a** OA-CAT Functional Difficulty Score Distribution: Baseline and Post-Exercise Training. **b** PROMIS® Physical Function CAT Score Distribution: Baseline and Post Exercise Training

OA-CAT FD and PROMIS® PF CAT scores were strongly correlated, with similar correlation coefficients at baseline and post-exercise training (0.65 and 0.69, respectively). OA-CAT FD and PROMIS® PF CAT score reliability values were similar at baseline (0.90 and 0.85, respectively) and post-exercise training (0.89 and 0.85, respectively). OA-CAT FD and PROMIS® PF CAT score distributions at baseline and post exercise training have similar distributions. (See Fig. 1a and Fig. 1b.)

The average number of items administered by the OA-CAT FD at baseline was 10.03 (SD = 2.62) and the time to complete was 2.76 (SD = 1.48) minutes. For the PROMIS® PF, an average of 4.14 (SD = 0.54) items were administered and it took 1.8 (SD = 0.66) minutes to complete.

### Sensitivity to change

**Fig. 2:**
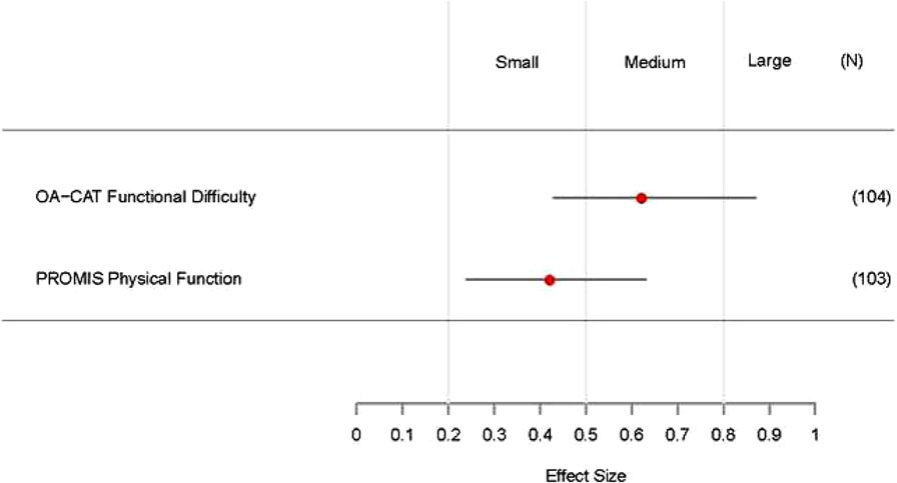
OA-CAT Functional Difficulty and PROMIS Physical Function Effect Size

The mean change of OA-CAT FD and the PROMIS® PF CAT scores from baseline to post-exercise training are presented in Table 3. Findings for both the OA-CAT FD and the PROMIS® PF CAT instruments show significant ESs, with the OA-CAT FD having a somewhat greater ES [0.62 (CI = 0.43, 0.87) compared to the PROMIS® PF CAT [(0.42 (CI = 0.24, 0.63). A statistical comparison of ES for the two measures is displayed in Fig. 2, demonstrating no significant differences in ES values between the OA-CAT FD and the PROMIS® PF CAT [95% CI = (− 0.43, 0.004)].

**Table 3:** Descriptive statistics of PROMIS® PF CAT and OA-CAT FD

Measure/Domain	Baseline (*N* = 120)	Post-Exercise Training (*N* = 104)	Effect Size (ES)
	Mean ± SD (Range)	Mean ± SD (Range)	ES (95%CI)
OA-CAT Functional Difficulty	49.53 ± 8.29 (33.35, 75.72)	54.75 ± 9.28 (24.17, 88.17)	0.62^a^ (0.43, 0.87)
PROMIS® Physical Function CAT	42.96 ± 5.72 (28.60, 60.70)	45.27 ± 5.67 (31.90, 57.20)	0.42^a^ (0.24, 0.63)

Note: ^a^ The 95% Confidence interval of the effect size (ES) does not contain 0, indicating that the change of score is significant

## Discussion

This study compared the relative ability of two PROs – OA-CAT FD, an OA-specific measure, and the PROMIS® PF CAT, a generic measure – in a sample of adults at risk of knee OA who completed in an exercise program designed to improve physical function. Results demonstrated that both instruments are able to detect change in function after participation in an exercise program known to have a beneficial effect on functional outcomes. The strong correlation between the two instruments provides evidence that they measure the same construct. Both scales were similar in terms of score reliability and score distribution. The OA-CAT administered more items and took longer to administer. This is likely due to the fact that OA-CAT includes a response option ‘Did not do the activity for reasons other than the arthritis in his/her legs.’ If this response is selected, the item is not included in the score determination. While more items are administered and it takes an additional minute to administer, the OA-CAT score may more accurately reflect the impact of arthritis on function. While no statistically significant difference in ES values was noted between the two instruments, trends suggest that the OA-CAT scale achieved a larger ES. These findings may indicate that the exercise intervention for more effective for improving function impacted by OA than it was for improving general physical function. Thus, the OA-CAT FD may be preferred for use in clinical trials, particularly when study power is somewhat compromised by sample size. Nonetheless, our study also supports use of the PROMIS® PF CAT to measure change in function among people with OA. PROMIS CAT PF mean score and range at baseline are similar to reports using the PROMIS PF short forms to assess function in a sample with symptomatic knee OA [28]. This study used Physical Function item bank v1.0 and newer, refined PROMIS® items banks include the addition of a Mobility v1.2 item bank focused on a range of activities from getting out of a bed or chair to activities such as running. This item bank may function better in a sample of persons with osteoarthritis than the Physical Function v1.0 item bank, which includes upper extremity activities and instrumental activities of daily living [29].

Performance-based measures were collected in this study, include the five-time-sit-to stand test (FTSST) shown to detect change in persons with OA completing a strength training program [30]. We examined FTSST baseline and post-exercise training scores to provide additional evidence that the exercise program resulted in change. The FTSST ES (0.5) is similar to the ES reported for the OA-CAT (0.62) and PROMIS (0.54), indicating that the exercise program produced similar change in a measure of performance. However, changes in strength may not translate into a similar change in function. In fact, correlations between FTSST times and OA-CAT and PROMIS scores were weak at baseline and post exercise training (− 0.21 to − 0.34), indicating that the FTSST measures a different construct.

Both the OA-CAT FD and the PROMIS® PF CATs provide the many advantages of contemporary measurement approaches compared to traditional fixed form PROs [31]. The primary difference between the two instruments is that the PROMIS® Physical Function CAT is a generic PRO measure and the OA-CAT is an osteoarthritis-specific PRO. Debates over the pros and cons of generic and disease-specific functional measures are frequently found in the literature [32, 33]. The generic instruments are universally applicable across diseases by measuring an overall condition, while the disease-specific instruments can usually tap into more and deeper disease-specific effects. Our study findings indicated that both the generic PRO measure and the osteoarthritis-specific PRO measure showed significant sensitivity to change and that both have potential usefulness.

Study limitations include a relatively small sample size and loss to follow-up. With a larger sample size, a significant difference in ESs may have been detected. Second, we did not compare the sensitivity to change of these instruments to other standard patient reported outcomes (e.g., Western Ontario and McMaster Universities Osteoarthritis Index (WOMAC)). Third, the exercise program was relatively brief and a longer duration could have resulted in greater ‘true’ change, enhancing the ability of these instruments to detect change. Fourth, we only have one follow-up data point and are unable to determine the sensitivity to change over longer periods of time which is often needed for chronic conditions such as OA.

## Conclusions

The investigation of the sensitivity to change for the OA-CAT FD and PROMIS® PF CAT suggests that both instruments have significant sensitivity to change in persons with OA. While no statistically significant difference was found in the sensitivity to change between the two instruments, the trends revealed that the OA-CAT achieved larger ES. Both instruments have potential usefulness in arthritis research.

## Abbreviations

CAT: Computerized Adaptive Test
CI: Confidence Intervals
ES: Cohen Effect Size
IRT: Item Response Theory
OA: Osteoarthritis
OA-CAT FD: Osteoarthritis Computerized Adaptive Test Functional Difficulty
PRO: Patient Reported Outcome
PROMIS® PF CAT: Patient Reported Outcomes Measurement Information System Physical Function scale
SD: Standard Deviation
SF-36: Short Form Health Survey
WOMAC: Western Ontario and McMaster Universities Osteoarthritis Index
